# L-Theanine Mitigates Acute Alcoholic Intestinal Injury by Activating the HIF-1 Signaling Pathway to Regulate the TLR4/NF-κB/HIF-1α Axis in Mice

**DOI:** 10.3390/nu17040720

**Published:** 2025-02-18

**Authors:** Simin Tan, Jiayou Gu, Jiahao Yang, Xuhui Dang, Kehong Liu, Zhihua Gong, Wenjun Xiao

**Affiliations:** 1 Key Lab of Tea Science of Ministry of Education, Hunan Agricultural University, Changsha 410128, China; 16673431436@163.com (S.T.); 18175550605@163.com (J.G.); 15616941136@163.com (J.Y.); 18991932097@163.com (X.D.); sumzzzz@163.com (K.L.); 2 National Research Center of Engineering Technology for Utilization of Botanical Functional Ingredients, Hunan Agricultural University, Changsha 410128, China

**Keywords:** alcohol consumption, L-theanine, acute alcohol intestinal injury, metabolomics, TLR4/NF-κB/HIF-1α axis

## Abstract

Background/Objectives: Acute alcohol consumption can cause intestinal dysfunction, whereas L-theanine (LTA) has shown the potential to support intestinal health. We explored L-theanine’s ability to protect against acute alcohol-induced injury. Methods: Male C57BL/6 mice were administered LTA for 28 d and then underwent acute alcohol intestinal injury modeling for 8 days. Results: The results revealed that LTA ameliorated alcohol-induced pathological damage in the duodenum and gut permeability, improved secretory immunoglobulin A (SIgA) content, and reduced oxidative stress, inflammatory markers, and serum lipopolysaccharide (LPS) content in mice. Furthermore, LTA restored the composition of the intestinal flora, increasing the abundance of *Alloprevotella*, *Candidatus_Saccharimonas*, *Muribaculum*, and *Prevotellaceae_UCG-001*. Additionally, LTA increased beneficial metabolites, such as oxyglutaric acid and L-ascorbic acid, in the HIF-1 pathway within the enrichment pathway. Further investigation into the HIF-1 signaling pathway identified up-regulation of claudin-1, HIF-1α, occludin, and ZO-1, and down-regulation of TLR4, PHD2, p65 NF-κB, TNF-α, and IFN-γ mRNA and protein levels. Conclusions: These results suggest that LTA may enhance the intestinal barrier by activating the HIF-1 signaling pathway to regulate the TLR4/NF-κB/HIF-1α axis, thereby reducing acute alcoholic intestinal injury.

## 1. Introduction

Alcohol serves as a significant cultural and social element worldwide. In recent years, the increasing prevalence of alcohol consumption has led to increasing health and socioeconomic concerns worldwide. While the primary prevention of alcohol abuse remains a cornerstone of public health interventions, complementary strategies are essential to address accidental alcoholism arising from unintentional overconsumption of alcohol by non-dependent individuals (e.g., cultural drinking rituals, drug interactions). Alcohol is predominantly absorbed in the intestine [1]. Both chronic and acute alcohol consumption can result in damage to the gastrointestinal mucosa and impair intestinal function, ultimately leading to intestinal injury [2]. Treating acute alcoholic intestinal injury presents a significant challenge due to its sudden onset and complex pathogenesis, which have yet to be fully understood.

Acute alcohol intake may cause structural damage to the intestinal tissue, which is often accompanied by the destruction of intestinal permeability and, thus, damage to the mechanical barrier of the intestinal epithelium. Intestinal microbiota imbalance, intestinal inflammatory response, and elevated levels of oxidative stress are also the main manifestations of intestinal injury [3]. Specifically, an imbalance in the gut microbiota due to alcohol can lead to an increase in the levels of the intestinal endotoxin lipopolysaccharide (LPS). LPS activates inflammation-related signaling pathways by mediating Toll-like receptor 4 (TLR4) pathways [4], such as the NF-κB pathways, leading to the release of inflammatory factors such as tumor necrosis factor-α (TNF-α), interleukin-1 (IL-1), and interferon-γ (IFN-γ) [5]. The production of inflammatory factors can damage the integrity of the intestinal barrier, alter the expression of intestinal compact-linking proteins (TJs), and impair intestinal function [6]. HIF-1α is a key transcription factor that is mainly activated under hypoxia conditions. It regulates the adaptive response of cells to hypoxia [7]. Proline hydroxylase (PHD) is a key enzyme that regulates the function of HIF-1α, enabling HIF-1α degradation at normal oxygen levels [8]. Chronic alcohol abuse or high-dose alcohol exposure may lead to decreased HIF-1α levels [9]. Moreover, loss of HIF-1α has been reported to exacerbate intestinal microbiota dysbiosis after alcohol exposure and to aggravate alcohol-related intestinal diseases [10].

Currently, the main treatment for acute alcohol-induced intestinal injury involves the administration of probiotics and antibiotics. However, these methods are plagued by issues such as drug resistance and determining safe ingestion doses [11]. In addition, intestinal injury due to acute alcohol is a sudden occurrence. Therefore, identifying a natural active ingredient that can be safely consumed in daily life—without toxic side effects—to prevent acute alcoholic intestinal injury holds considerable practical importance. Natural products sourced from plants and herbs generally exhibit low toxicity [12]. Research has indicated that a phenol-rich extract from cactus exhibits anti-inflammatory properties that alleviate colitis; however, further exploration is required to determine the safe dosage of this extract [13]. LTA is a non-toxic characteristic amino acid, and the technology for extracting LTA from tea is now very mature [14]. L-theanine, which is generally recognized as safe (GRAS) [15], is easily absorbed by the intestine [16]. It is associated with various biological activities, including immunity enhancement, antioxidant effects, and anti-inflammatory properties [17]. Our research group previously demonstrated that LTA exerts protective effects on the intestinal barrier and morphology [18]. In addition, studies have shown that LTA can alleviate liver damage caused by acute alcohol intake in mice by accelerating alcohol metabolism and regulating the NF-κB pathway [19]. Although previous studies have established the general protective effects of LTA on gut health through antioxidant and anti-inflammatory mechanisms, its specific interaction with the HIF-1 signaling pathway in the context of alcohol-induced intestinal injury remains unexplored. Notably, the regulatory crosstalk between HIF-1α and the TLR4/NF-κB axis under ethanol exposure has not been systematically investigated in prior research on LTA. Therefore, we investigated the protective effects of LTA on acute alcoholic intestinal injury in C57BL/6 mice and elucidated the possible mechanisms of these effects using gut microbiome and metabolome sequencing. This study aimed to establish a scientific basis for the formulation of LTA as a dietary supplement, emphasizing its potential role in preventing acute intestinal injury associated with alcohol consumption.

## 2. Materials and Methods

### 2.1. Reagents

LTA (98% purity) was obtained from Hunan Sanfu Biotechnology Co., Ltd. (Changsha, China). Lieber–DeCarli diet was obtained from Hunan Slack Jingda Laboratory Animal Co., Ltd. (Changsha, China).

### 2.2. Animals and Treatments

Fifty SPF grade C57BL/6 male mice (6 weeks old, weighting 20–22 g) were obtained from Hunan Slack Jingda Laboratory Animal Co., Ltd. (Changsha, China; license number SCXK [Xiang] 2023–0004). All animal experiments were conducted in accordance with the National Institutes of Health Guidelines for the Care and Use of Laboratory Animals (National Institutes of Health Publication No. 8023, revised 1978) and the Animal Health and Ethics Committee of Hunan Agricultural University (Approval No. 2022 No. 11, 1 January 2023–31 December 2026). The mice were housed in a controlled environment with a 12 h light–dark cycle, humidity set at 55% ± 5%, and temperature maintained at 25 °C ± 2 °C.

### 2.3. Acute Alcoholic Intestinal Injury Model

After 1 week of liquid diet adaptation, the acute alcoholic intestinal injury model construction method is referred to as the Lieber–Decarli and Gao–Binge model [20,21] and is slightly modified. Specifically, the mice were fed a Lieber–DeCarli diet containing 5% (vol/vol) ethanol (5%EtOH diet) for 7 days, followed by a single dose of ethanol (5 g/kg body weight) on day 8, followed by euthanasia at 9 h.

### 2.4. Treatments

**Figure 1:**
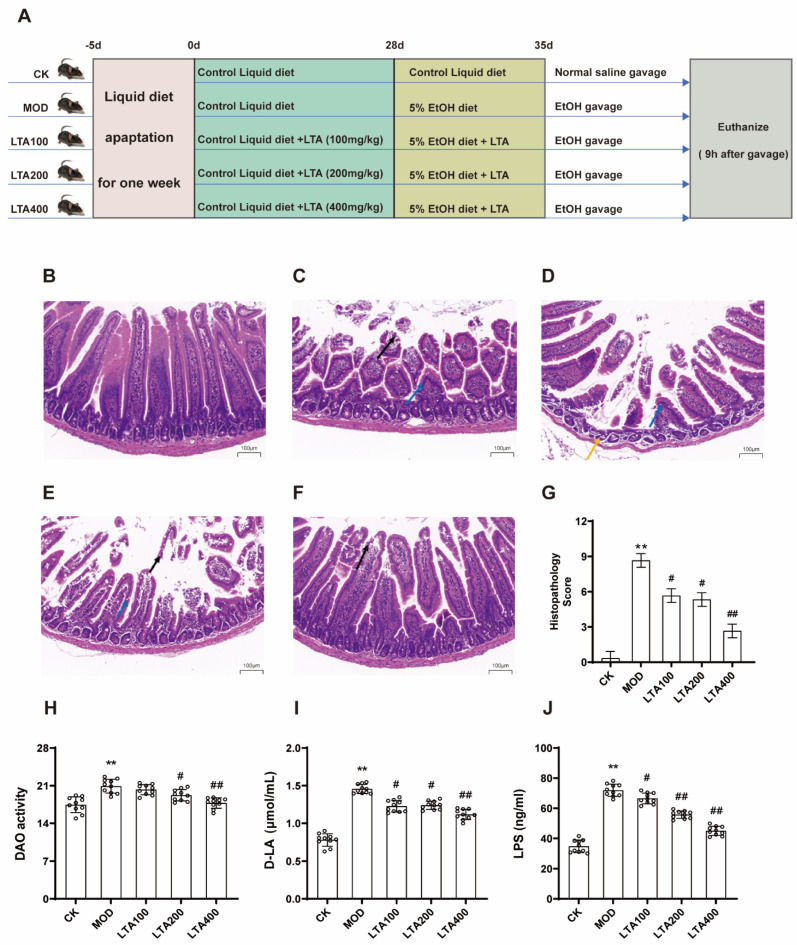
Experimental design flow chart and effect of LTA on duodenum tissue sections and intestinal permeability in mice. (**A**) Schematic representation of animal experiments (n = 10). (**B**–**F**) The H&E tissue sections were CK, MOD, LTA100, LTA200, and LTA400 groups, respectively (H&E, ×20, n = 3). Villi apical autolysis (blue arrow), Intestinal villi epithelial cells shed (black arrow), and lymphoid tissue hyperplasia (yellow arrow). (**G**) Intestinal histological score (n = 3). (**H**) LTA effect on serum DAO activity in mice (n = 10). (**I**) LTA effect on serum D-LA content in mice (n = 10). (**J**) LTA effect on serum LPS content in mice (n = 10). Compared with CK, **: *p* < 0.01; compared with MOD, #: *p* < 0.05, ##: *p* < 0.01, Values are reported as mean ± SD.

After 1 week of liquid diet acclimation, the mice were randomly divided into five groups: normal control (CK), model (MOD), low-dose LTA (LTA100), medium-dose LTA (LTA200), and high-dose LTA (LTA400), with 10 mice per group. Mice in the LTA100, LTA200, and LTA400 groups were administered LTA at doses of 100, 200, and 400 mg·kg^−1^·d^−1^ added to the Lieber–DeCarli diet from 9:00 a.m. to 10:00 a.m. daily for 28 days. CK and MOD mice were fed a normal Lieber–DeCarli diet. LTA supplementation was calculated according to the body weight and feed intake of each group of mice (body weight and feed intake of mice in each group are shown in Figure S1). The dose of LTA administered was determined based on a previous study [17,19]. Using a conversion factor of 0.11 to extrapolate from mice to humans, the doses of 100, 200, and 400 mg·kg^−1^·d^−1^ in mice correspond to human equivalent doses of 11, 22, and 44 mg·kg^−1^·d^−1^, which equate to approximately 660, 1320, and 2640 mg per day for an adult. According to the literature, supplementation of 300 mg·kg^−1^·d^−1^ LTA for 16 weeks in C57BL/6 mice is safe [22], suggesting that the dosage in this trial is reasonable. Subsequently, the model of acute alcoholic intestinal injury described above was followed for 8 days, and the LTA dose group was supplemented with alcohol and corresponding doses of LTA. The details of the animal experiments are shown in Figure 1A. No adverse events occurred during the experimental period. On the last day, the mice were fasted for 12 h after feeding, and fresh feces were collected for preservation. The mice were then anesthetized with 2% pentobarbital, and blood samples were collected from the mice through the retro-orbital sinus method. The intestinal contents and various intestinal tissues were collected, and blood was collected and centrifuged at 4 °C and 3000 rpm for 10 min.

### 2.5. Histopathological Assessment

The extracted intestinal tissue was fixed in a 10% (*v*/*v*) formaldehyde solution, and H&E staining was conducted at Pinofey Biotechnology Co., Ltd. (Wuhan, China). Histological scoring criteria, slightly adjusted from previous descriptions [23], were applied (Table S1).

### 2.6. Biochemical Analysis of Serum and Intestinal Tissue Samples

Serum levels of Diamine oxidase (DAO, A088-1-1), D-lactic acid (D-LA, H263), and LPS (AJ-3418) were determined by using commercial kits (Nanjing Jiancheng Biology Engineering Institute, Nanjing, China).

Small intestinal tissue levels of malondialdehyde (MDA A003-1-2), glutathione peroxidase-px (GSH-Px A005-1-2), superoxide dismutase (SOD A001-2-2), catalase (CAT A007-1-1), and SIGA (CSB-E08413m) were determined by using kits (Nanjing Jiancheng Biology Engineering Institute, Nanjing, China).

Small intestinal tissue levels of tumor necrosis factor-α (TNF-α P6335), interleukin-10 (IL-10 P6290), interleukin-6 (IL-6 P6276), and interleukin-1β (IL-1β P6245) were determined by using kits (Shanghai Biyontime Biotechnology Co., Ltd., Shanghai, China).

### 2.7. Gut Microbiota Determination

Six mice were randomly selected from each group and fresh feces were collected using sterile tweezers and centrifuge tubes before euthanizing the mice. Mice feces were used to amplify the bacterial V3–V4 variable region via PCR, and genomic DNA was extracted using the E.Z.N.A genomic DNA extraction kit (Omega Bio-Tek, Norcross, GA, USA). Sequencing was performed using Illumina’s Miseq PE300 platform. UPARSE software (version 7.1) performs OTU clustering on sequences and sets OUT sequence similarity to 0.97. Each sequence is classified and annotated using the RDP classifier and compared to the Silva database (SSU123) with a comparison threshold of 0.7.

### 2.8. LC/MS-Based Gut Metabolomics

Sample pretreatment according to the previously reported pretreatment procedure [24]. LC-MS analysis was performed using a Waters ACQUITY UPLC I-Class instrument coupled to a Thermo QE ultra-high-performance liquid chromatography-mass spectrometer. Differential metabolites were screened using OPLS-DA and *t*-tests, with the screening criteria set at variable importance in projection > 1 and *p* < 0.05. Metabolic pathway enrichment analysis of the identified metabolites was conducted using MetaboAnalyst 5.0 and the Kyoto Encyclopedia of Genes and Genomes (KEGG) database.

### 2.9. Quantitative Real-Time PCR

Total RNA was extracted from small intestinal tissues using the RNA Easy Fast kit (DP451, Beijing Tiangen Biotech Co., Ltd., Beijing, China). Subsequently, cDNA synthesis was performed using the FastKing gDNA kit (KR118) and the RT SuperMix (FP209). The subsequent detection and analysis methods were based on previously described methods [18]. β-actin was used as the internal reference. Primer sequence information is provided in the Table S2.

### 2.10. Western Blotting

Protein extracts from intestinal tissues were prepared and analyzed as previously described [25]. The primary antibodies information are as follows: TLR4 (AF7017, Rab, 1:1000, affinity, Changsha, China), p65 NF-κB (10745-1-AP, Rab, 1:3000, Proteintech, Wuhan, China), IFN-γ (DF6045, Rab, 1:4000, affinity, Changsha, China), occludin (bsm-60763M, Rab, 1:1000, bipss, Beijing, China), PHD2(66589-1-Ig, Rab, 1:1000, Proteintech, Wuhan, China), HIF-1α(AF1009, Rab, 1:000, affinity, Changsha, China), ZO-1(21773-1-AP, Rab, 1:1000, Proteintech, Wuhan, China), claudin-1 (AF0127, Rab, 1:1000, affinity, Changsha, China), TNF-α(AF7014, Rab, 1:1000, affinity, Changsha, China), β-actin (81115-1-RR; Rab, 1:10,000, Proteintech, Wuhan, China), and GAPDH (60004-1-Ig, Rab, 1:10,000, Proteintech, Wuhan, China). The secondary antibody information is as follows: HRP-Goat anti-Rabbit (bs-0295G, Rab, 1:5000, Abiowell, Changsha, China).

### 2.11. Statistical Analysis

Throughout sample processing and data analysis, researchers involved in histopathological scoring, biochemical assays, sequencing, and statistical evaluation were blinded to group assignments. All samples were labeled with randomized codes, and group information was only revealed after the completion of data collection and preliminary analysis to minimize observer bias. IBM SPSS (version 19.0, SPSS, Inc., Chicago, IL, USA) was used for statistical analysis. For samples that pass the homogeneity of the variance test, one-way ANOVA with Duncan is performed. In contrast, for those samples that did not satisfy the homogeneity of variance requirement, significance was determined using the Tamhane T2 test. GraphPad Prism 8.0.2 was used to draw graphs. Tissue sections were observed and analyzed using CaseViewer (Shanghai, China). Variance homogeneity: Levene’s test (*p* < 0.05) determined the variance heterogeneity of variables, necessitating Tamhane T2 post-hoc tests. The experimental results were expressed as mean ± SD. Statistical significance was set at *p* < 0.05.

## 3. Results

### 3.1. LTA Attenuates Duodenal Pathology and Intestinal Permeability Injury in Mice with Acute Alcoholic Intestinal Injury

Histological sections of the mouse duodenum indicated that the MOD group exhibited shortened intestinal villi (blue arrow) and epithelial cell shedding from the intestinal villi (black arrow, Figure 1C). Moreover, the histological scores in the MOD group were significantly higher than those in the CK group (Figure 1G, *p* < 0.01; see Table S1 for histological scoring criteria). However, after different doses of LTA were administered, the alcohol-induced damage was alleviated. Compared with that in the MOD group, the histological score in the LTA400 group was significantly reduced (Figure 1G, *p* < 0.01).

Compared with the CK group, the serum levels of DAO, D-LA, and LPS were increased in the MOD group (Figure 1H–J, *p* < 0.01). In addition, compared with that in the MOD group, DAO, D-LA, and LPS levels in all LTA groups were decreased, and the reduction was most significant in the LTA400 group (*p* < 0.01).

### 3.2. LTA Improved Intestinal Inflammatory Injury and Oxidative Stress in Mice with Acute Alcoholic Intestinal Injury

**Figure 2:**
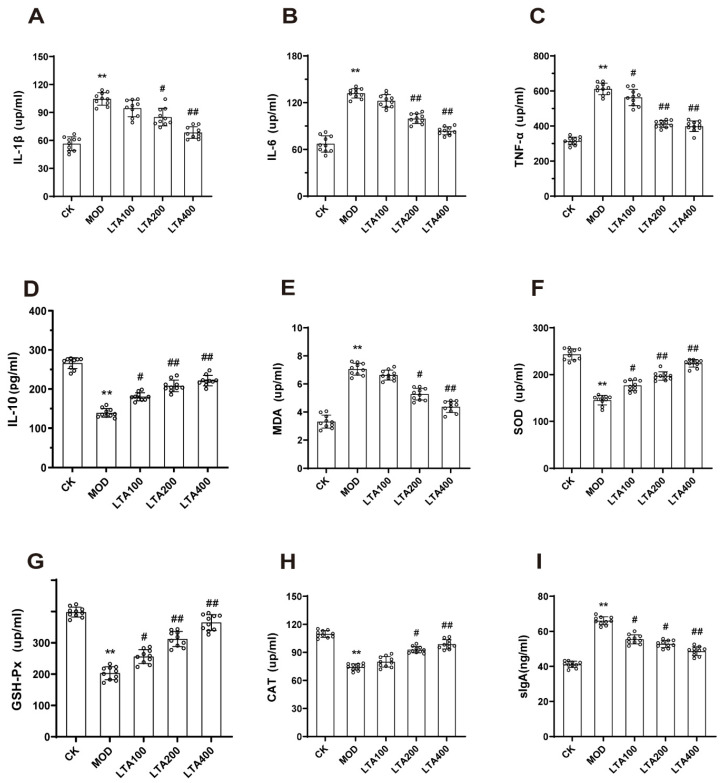
Effects of LTA on inflammatory factors, antioxidant capacity, and SIgA levels in intestinal tissue of mice (n = 10). (**A**) IL-1β, (**B**) IL-6, (**C**) TNF-α, (**D**) IL-10, (**E**) MDA, (**F**) SOD, (**G**) GSH-Px, (**H**) CAT, (**I**) SIgA levels. Compared with CK, **: *p* < 0.01; compared with MOD, #: *p* < 0.05, ##: *p* < 0.01. Values are reported as mean ± SD.

Next, the levels of intestinal inflammatory factors and oxidative stress in mice with acute alcohol-induced intestinal injury were evaluated. Compared with the CK group, the TNF-α, IL-1β, and IL-6 levels (Figure 2A–C, *p* < 0.01) were increased in the MOD group, and the IL-10 levels (Figure 2D, *p* < 0.01) were decreased in the MOD group.

Compared to the MOD group, TNF-α levels in small intestine tissues of the LTA100 group were decreased (Figure 2C, *p* < 0.05), while IL-10 levels increased (Figure 2D, *p* < 0.05). In the LTA200 group, IL-1β (Figure 2A, *p* < 0.05), IL-6, and TNF-α (Figure 2B–C, *p* < 0.01) levels were lower than those in the MOD group, with an increase in IL-10 levels (Figure 2D, *p* < 0.01). Similarly, in the LTA400 group, IL-1β, IL-6, and TNF-α levels were decreased compared to the MOD group (Figure 2A–C, *p* < 0.01), while IL-10 levels were increased (Figure 2D, *p* < 0.01).

Compared with the CK group, the MDA level was increased and SOD, GSH-PX, and CAT levels were decreased in the MOD group (Figure 2E–H, *p* < 0.01).

Compared to the MOD group, all LTA dosage groups effectively reversed the alterations in the aforementioned indices. Notably, compared to the MOD group, the LTA400 group exhibited the most pronounced effect, demonstrating a significant reduction in MDA levels in the small intestine (Figure 2E, *p* < 0.01), while simultaneously increasing SOD, GSH-Px, and CAT levels (Figure 2F–H, *p* < 0.01).

We assessed intestinal SIgA levels and the results indicated that, compared with that in the CK group, the SIgA level in the MOD group was significantly reduced (Figure 2I, *p* < 0.01). Compared with the MOD group, the SIgA level in the LTA100 and LTA200 groups was reduced (*p* < 0.05), and, in the LTA400 group, was significantly reduced (*p* < 0.01).

In summary, LTA exerts protective effects against intestinal injury caused by acute alcohol consumption. The protective effect was the strongest in the LTA400 group. Therefore, this group was selected for the subsequent experiments.

### 3.3. LTA Regulates the Intestinal Flora Structure in Mice with Acute Alcoholic Intestinal Injury

**Figure 3:**
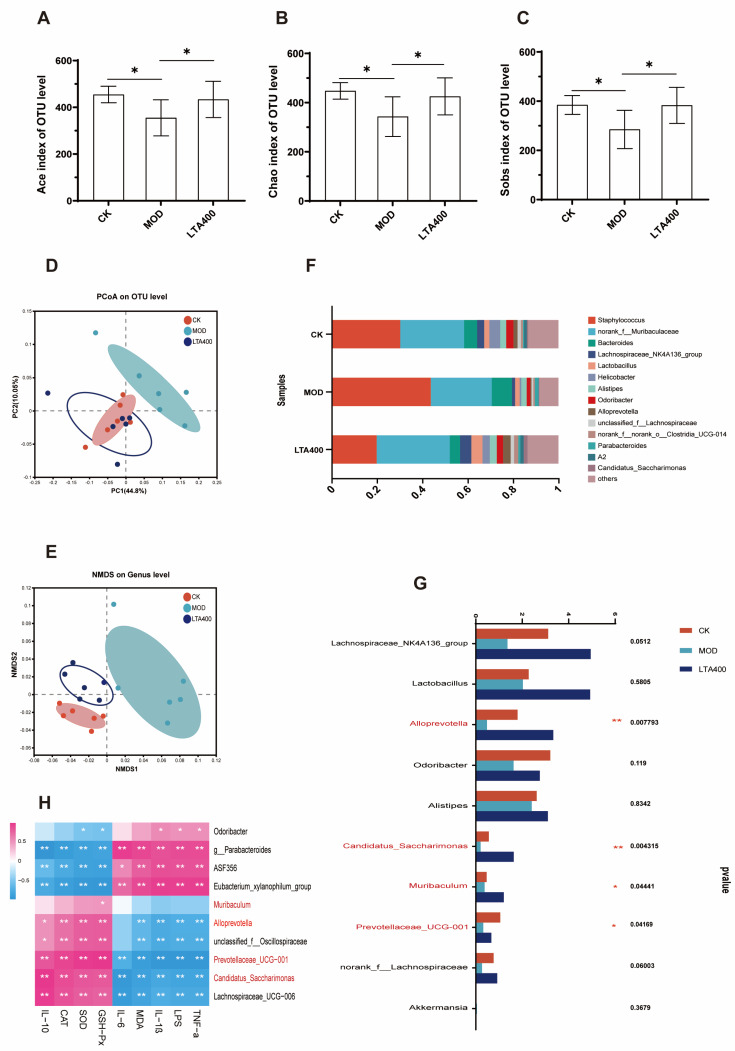
Effects of LTA on gut microbiota in mice with acute alcohol intake (n = 6). (**A**) Ace index of OUT level. (**B**) Chao index of OUT level. (**C**) Sobs index of OUT level. (**D**) Principal component analysis (PCoA) of OUT level. (**E**) Nonmetric multidimensional scale (NMDS) analysis of genus level. (**F**) Distribution of community composition at the genus level. (**G**) Significance test for differences between groups. (**H**) Spearman correlation analysis of the abundance of specific genera in fecal flora with serum/intestinal parameters. *: *p* < 0.05, **: *p* < 0.01. Values are reported as mean ± SD.

This study evaluated α-diversity by using Ace, Chao, and Sobs indices. The results showed that the Ace, Chao, and Sobs indices of the MOD group were lower than that of the CK group (Figure 3A–C, *p* < 0.05). Compared with the MOD group, the index of Ace, Chao, and Sobs increased significantly after LTA intervention (Figure 3A–C, *p* < 0.05). Nonmetric multidimensional scaling analysis (NMDS) and principal coordinate analysis (PCoA) were utilized to conduct β-diversity analysis, investigating the overall structural alterations in gut microbiota following LTA intervention. The score maps for PCoA and NMDS (Figure 3D,E) revealed a significant separation between the experimental groups. Notably, the structure of the intestinal flora tended to move to the CK group, indicating that the addition of LTA could, to a certain extent, restore the intestinal flora damaged by acute alcohol intake. Analysis of the community composition revealed that, at the genus level (Figure 3F), the intestinal flora mainly comprised *Staphylococcus* (30.29%), *norank_f_Muribaculaceae* (28.17%), *Bacteroides* (5.7%), and *Lachnospiraceae_NK4A136_*group (3.1%). In addition, significance tests between the groups revealed that, compared with the CK group, alcohol intake significantly decreased the abundance of *Alloprevotella*, *Candidatus_Saccharimonas*, *Muribaculum*, and *Prevotellaceae_UCG-001* (Figure 3G, *p* < 0.05). Compared with the MOD group, LTA intervention increased the abundance of *Alloprevotella*, *Candidatus_Saccharimonas*, *Muribaculum*, and *Prevotellaceae_UCG-001* in the intestine (Figure 3G, *p* < 0.05). Spearman heat maps were used to investigate potential correlations between distinct gut microbiota and biochemical parameters. As presented in Figure 3H, *Alloprevotella*, *Muribaculum*, *Candidatus_Saccharimonas*, and *Prevotellaceae_UCG-001* were significantly negatively correlated with proinflammatory factors (*p* < 0.01), MDA, and LPS. These genera were also positively correlated with IL-10 and the antioxidant enzymes SOD, GSH-Px, and CAT (*p* < 0.01).

### 3.4. LTA Maintains Intestinal Metabolic Balance in Mice with Acute Alcoholic Intestinal Injury

**Figure 4:**
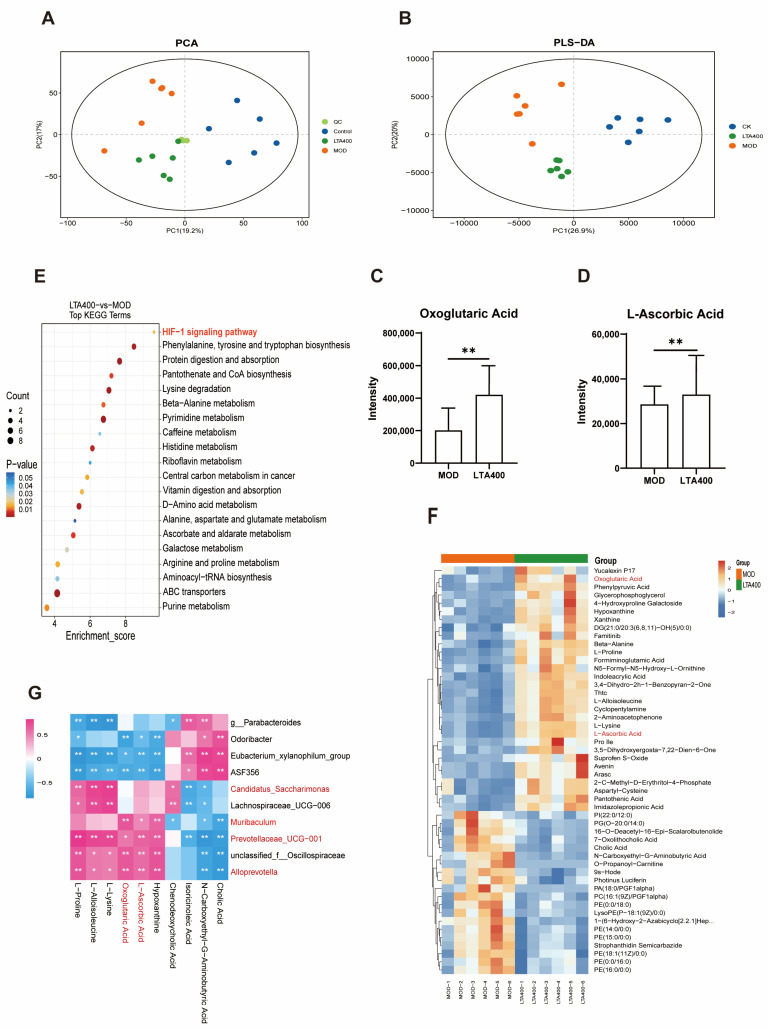
Effects of LTA on metabolites of intestinal contents in mice (n = 6). (**A**) PCoA score plot. (**B**) PLS-DA score plot. (**C**) Oxoglutaric acid levels. (**D**) L-ascorbic acid levels. (**E**) KEGG pathway classification (LTA400 vs. MOD). (**F**) Heat map showing the expression of the top 50 differential metabolites (LTA400 vs. MOD). (**G**) Spearman correlation analysis of abundances of specific genera in fecal flora and specific intestinal metabolites. *: *p* < 0.05, **: *p* < 0.01. Data are represented as mean ± SD.

Metabolomic techniques were used to analyze the effects of LTA on intestinal metabolites in mice. The clear clustering of the QC samples in the PCoA diagram suggested the stability and accuracy of the experiment (Figure 4A). The PLS-DA results revealed significant differences between the CK, MOD, and LTA400 groups, indicating notable variations in metabolites across each group (Figure 4B). Heat maps of the relative abundance of significant metabolites in the TOP50 group revealed differences in the levels of some metabolites between the MOD and LTA400 groups (Figure 4F); the metabolites with the most significant differential expression were amino acids, peptides, fatty acids, carbohydrates, and derivatives. LTA significantly increased the levels of oxoglutaric acid and L-ascorbic acid (Figure 4C,D, *p* < 0.01). Metabolic pathway enrichment analysis of the intestinal metabolites was performed using the KEGG database to identify the key metabolic pathways that were affected. The following processes were enriched according to the results of KEGG analysis: HIF-1 signaling pathway, protein digestion and absorption, amino acid synthesis catabolism, ABC transport signaling pathway, vitamin digestion and absorption, and other related signaling pathways. In comparison to the MOD group, intervention with LTA400 significantly activated the HIF-1 signaling pathway (Figure 4E, *p* < 0.05). The Spearman correlation coefficient was calculated to investigate the relationship between intestinal flora and intestinal metabolites. *Alloprevotella*, *Muribaculum*, and *Prevotellaceae_UCG-001* were significantly positively correlated with oxoglutaric acid and L-ascorbic acid (Figure 4G, *p* < 0.01).

### 3.5. Activation of the HIF-1 Signaling Pathway to Regulate the TLR4/NF-κB/HIF-1α Axis by LTA and the Consequent Enhancement of the Intestinal Mechanical and Immune Barrier in Mice

**Figure 5:**
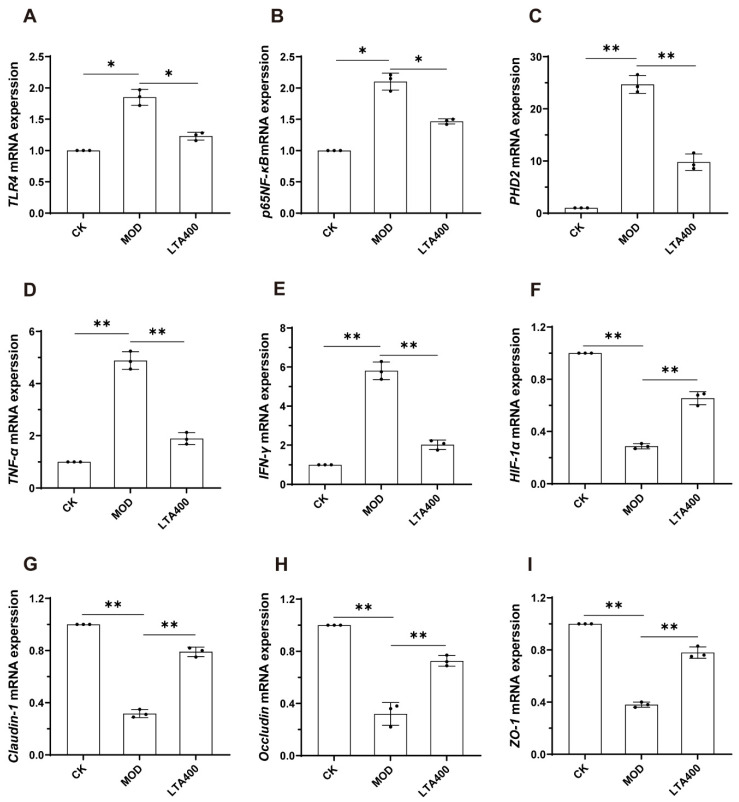
Effect of LTA on mRNA expression associated with HIF-1 signaling and intestinal injury in mice (n = 3). (**A**) *TLR4*. (**B**) *NF-κB*. (**C**) *PHD2*. (**D**) *TNF-α*. (**E**) *IFN-γ*. (**F**) *HIF-1α*. (**G**) *Claudin-1*. (**H**) *Occludin*. (**I**) *ZO-1*. *: *p* < 0.05, **: *p* < 0.01. Values are reported as mean ± SD.

**Figure 6:**
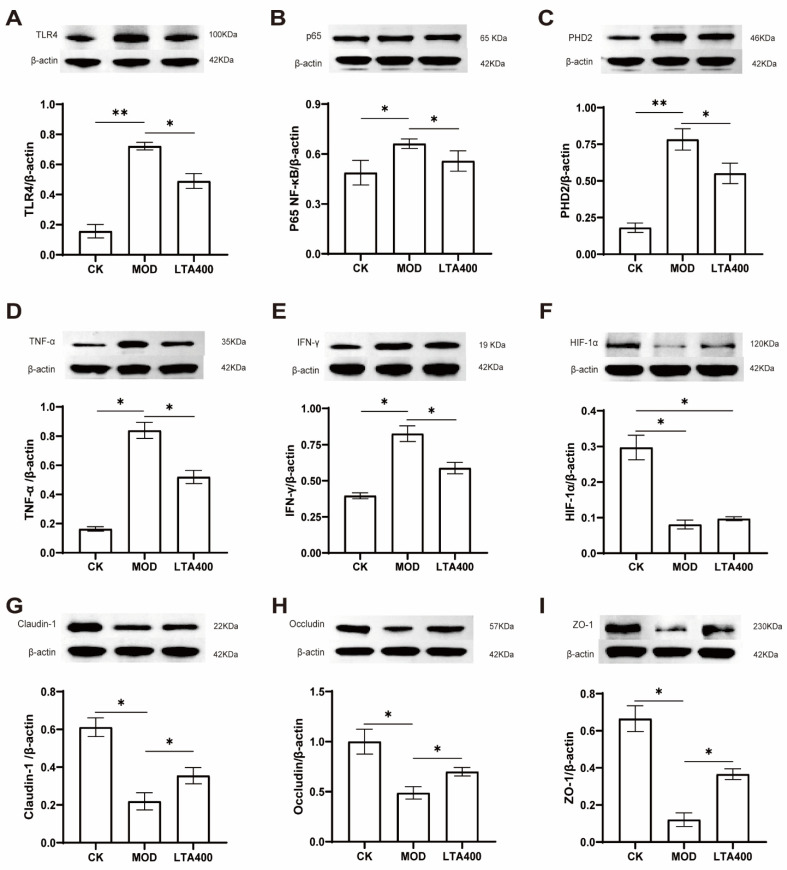
Effect of LTA on protein expression associated with HIF-1 signaling and intestinal injury in mice (*n* = 3). (**A**) TLR4. (**B**) NF-κB. (**C**) PHD2. (**D**) TNF-α. (**E**) TNF-α. (**F**) HIF-1α. (**G**) Claudin-1. (**H**) Occludin. (**I)** ZO-1. *: *p* < 0.05, **: *p* < 0.01. Values are reported as mean ± SD.

To verify the reliability of our results, the mRNA and protein expression levels of TLR4, NF-κB, HIF-1α, PHD, TNF-α, IFN-γ, claudin-1, occludin, and ZO-1 were detected with reference to the HIF-1 metabolic pathway enriched in the KEGG analysis of the intestinal metabolome data (Figure 5 and Figure 6).

Compared to the CK group, the mRNA expression levels of *TLR4*, *NF-κB*, *PHD2*, *TNF-α*, and *IFN-γ* in the small intestine of the MOD group were significantly up-regulated (Figure 5A–E, *p* < 0.05). Notably, when compared to the MOD group, the mRNA expression levels of *TLR4*, *NF-κB*, *PHD2*, *TNF-α*, and *IFN-γ* in small intestinal tissue were significantly down-regulated after LTA intervention (Figure 5A–E, *p* < 0.05). In addition, compared with the CK group, the mRNA expression levels of *HIF-1α*, *claudin-1*, *occludin*, and *ZO-1* in small intestine tissues of the MOD group were significantly down-regulated (Figure 5F–I, *p* < 0.05). Compared with the MOD group, the mRNA expression levels of *HIF-1α*, *claudin-1*, *occludin*, and *ZO-1* in small intestinal tissue were significantly up-regulated after LTA intervention (Figure 5F–I, *p* < 0.05).

The results of protein detection indicated that, in comparison to the CK group, the expression levels of TLR4, NF-κB, PHD2, TNF-α, and IFN-γ were significantly up-regulated in the MOD group; conversely, the expression levels of HIF-1α, claudin-1, occludin, and ZO-1 were markedly down-regulated (Figure 6A–E, *p* < 0.05). Notably, when compared to the MOD group, LTA intervention led to a significant down-regulation of TLR4, NF-κB, PHD2, TNF-α, and IFN-γ protein expression levels while simultaneously resulting in a significant up-regulation of claudin-1, occludin, and ZO-1 protein levels (Figure 6F–I, *p* < 0.05).

## 4. Discussion

Global epidemiological studies highlight geographic disparities in alcohol-induced gut injury mechanisms. For instance, Western populations exhibit higher susceptibility to ethanol-induced mucosal barrier disruption due to dietary patterns rich in saturated fats [26], while Asian cohorts show microbiome-mediated metabolic vulnerabilities linked to genetic polymorphisms in alcohol dehydrogenase [27]. The gut is not only the main site for alcohol absorption but also plays a key role in digestion and nutrient absorption. Accidental alcohol poisoning can lead to intestinal dysfunction and affect overall health. LTA can regulate intestinal flora and aid in maintaining intestinal mucosal integrity [18]. Therefore, in this study, non-targeted metabolomics and microbial sequencing were used to investigate intestinal damage caused by acute alcohol consumption and the protective effects of LTA administration.

The success of the animal experiment model is the basis of this study. Although there are no direct reports on intestinal injury caused by acute alcohol, from the perspective of intestinal injury, intestinal pathological injury, intestinal permeability, intestinal inflammatory factors, and oxidative stress levels are the markers for evaluating intestinal injury [28,29]. In this study, the above markers were used to confirm whether the acute alcoholic intestinal injury model was established or not. The results demonstrated that acute alcohol consumption led to pathological duodenal injury, increased intestinal permeability, and exacerbated intestinal inflammation and oxidative stress. These findings indicate that acute alcohol intake induces intestinal injury in mice and validates the effectiveness of our modeling approach for acute alcoholic intestinal injury [30].

To investigate the protective effects of LTA against acute alcohol-induced intestinal injury, we treated mice with different doses of LTA. Our findings indicated that LTA reduced duodenal pathology scores in acute alcoholic mice. Similar effects of LTA were also observed in heat-stress-treated mice. DAO activity, D-LA levels, and LPS levels in the blood are useful markers for evaluating intestinal permeability [31,32]. Notably, LTA can improve the intestinal permeability induced by heat stress or colitis by reducing DAO activity, D-LA levels, and serum LPS levels [18,33]. In the current study, a similar phenomenon was observed regarding the preventive effects of LTA against acute alcoholic intestinal injury.

Some studies have reported that alcohol consumption increases MDA levels in intestinal tissue while decreasing SOD, GSH-Px, and CAT levels [34,35]. Our study observed similar effects; notably, LTA intervention reduced ROS and MDA levels in intestinal tissue and increased SOD, GSH-Px, and CAT levels. The levels of intestinal inflammatory factors are important indicators of intestinal health [36,37]. Therefore, we assessed these. Specifically, LTA was found to decrease the levels of pro-inflammatory cytokines (IL-1β, IL-6, TNF-α) while simultaneously increasing the levels of the anti-inflammatory cytokine IL-10 in mice with alcoholic intestinal injury. Notably, LTA also showed the same effect in mice with ulcerative colitis [38,39]. Collectively, LTA displayed a protective effect against acute alcohol-induced intestinal injury, with the LTA400 group showing the best protective effect. Collectively, LTA displayed a protective effect against acute alcohol-induced intestinal injury, with the LTA400 group showing the best protective effect.

Alcohol consumption can affect the composition of intestinal flora and lead to intestinal microbiota dysbiosis, which is a major factor in alcohol-induced intestinal injury [40]. The richness and diversity of microbial communities are often associated with a healthy gut [41]. In the present study, we found that LTA alleviated acute alcohol-induced intestinal microbiota dysbiosis. High doses of LTA significantly reduced the Ace, Chao, and Sobs indices and maintained the stability of the intestinal flora, consistent with previous results [18]. Furthermore, LTA intervention significantly increased the relative abundance of *Alloprevotella*, *Candidatus_Saccharimonas*, *Muribaculum*, and *Prevotellaceae_UCG-001* and improved the intestinal flora disturbance induced by alcohol intake in mice. *Alloprevotella*, a probiotic, plays a key regulatory role in maintaining the intestinal barrier function [42]. *Candidatus_Saccharimonas*, *Muribaculum*, and *Prevotellaceae_UCG-001* have been shown to reduce intestinal inflammation [43,44,45]. Notably, intestinal inflammation leads to an increase in LPS levels, which can disrupt intestinal barrier function [46]. In the current study, heat maps of microbial flora and biochemical parameters indicated that the abundance of the beneficial bacteria *Alloprevotella*, *Candidatus_Saccharimonas*, *Muribaculum*, and *Prevotellaceae_UCG-001* was positively associated with anti-inflammatory factors, antioxidant capacity, and serum LPS levels. Therefore, we hypothesized that LTA could improve intestinal microbiota disruption caused by alcohol intake, thereby facilitating the return of LPS to normal levels and alleviating intestinal oxidative stress and inflammation.

We analyzed intestinal content metabolism and discovered that LTA increased oxoglutaric acid and L-ascorbic acid levels and activated the HIF-1 signaling pathway. Notably, supplemental oxoglutaric acid can enhance intestinal antioxidant capacity, reduce intestinal inflammation, and promote intestinal immune response and barrier function [47]. Moreover, L-ascorbic acid, also known as vitamin C, regulates intestinal flora and enhances intestinal antioxidant capacity [48]. Therefore, we hypothesize that LTA activates HIF-1 signaling by restoring the composition of the intestinal flora, regulating LPS levels, and promoting oxygen glutaric acid and L-ascorbic acid metabolism.

The HIF-1 signaling pathway is critical for adapting to hypoxia and regulating energy metabolism in animal cells. HIF-1 is a heterodimer that is mainly composed of HIF-1α and HIF-1β [49]. Notably, HIF-1α contributes to the maintenance of intestinal flora homeostasis and defense against pathogens. Specifically, it plays a key role in maintaining the integrity of the tight connections of intestinal epithelial cells [50,51]. Therefore, we conducted a detailed study of the HIF-1 signaling pathway and generated a pathway map using KEGG enrichment analysis of intestinal metabolomic data (see Supplementary Materials), involving LPS, TLR4, NF-κB, HIF-1α, and PHD.

TLR4 is a transmembrane receptor that recognizes LPS and activates immune responses [52]. In our study, mRNA and protein expression levels of TLR4 were upregulated in the MOD group, subsequently increasing mRNA and protein expression of NF-κB, TNF-α, and IFN-γ. This response may be attributed to intestinal flora disturbances and elevated LPS production induced by alcohol intake, leading to heightened TLR4 expression in mice. Moreover, LTA treatment normalized intestinal SIgA levels, possibly through NF-κB activation [53], regulating the secretion of TNF-α, IFN-γ, and SIgA, thereby mitigating intestinal inflammation and immune response disruption.

PHD is an enzyme that can promote ubiquitination and degradation of the HIF-1α protein through hydroxylation, with PHD2 exhibiting superior catalytic activity toward HIF-1α compared to PHD1 and PHD2 [54]. However, the relationship between activation of NF-κB and HIF-1α remains unclear [55]. In the current study, activation of NF-κB in mice with alcohol ingestion was accompanied by significant decreases in mRNA and protein levels of HIF-1α, consistent with the results of a previous study [56].

Studies have shown that HIF-1a can promote the proliferation and repair of intestinal epithelial cells and enhance the expression of intestinal tight junction protein to protect the intestine [10]. In the current study, acute alcohol intake downregulated the expression level of HIF-1α, claudin-1, occludin, and ZO-1 mRNA and protein in the intestine of mice while up-regulating the expression levels of PHD2 mRNA and protein. The results indicate that alcohol reduces HIF-1α expression, thereby affecting TJs and disrupting intestinal mucosal function. In this study, the repair effect of LTA on the mechanical intestinal barrier in mice was also observed in the mode of heat stress [18]. However, LTA treatment significantly restored the expression of these genes and proteins to normal levels. Although mRNA expression of HIF-1α showed a significant increase, protein expression exhibited an upward trend without reaching statistical significance. This discrepancy may be attributed to the regulatory role of mRNA in modulating protein activity during translation. Pharmacological HIF-1 activators such as dimethyl allyl glycine (DMOG) have been shown to show similar protective effects [57]; LTA represents a unique dietary compound that achieves comparable pathway modulation through natural metabolic intermediates like oxyglutaric acid and L-ascorbic acid, suggesting distinct advantages.

In summary, LTA restored intestinal tissue structure, reduced intestinal permeability, and reduced intestinal inflammation and oxidative stress levels in mice with acute alcoholic intestinal injury. In addition, LTA regulates the intestinal flora and the TLR4/NF-κB/HIF-1α axis of the HIF-1 signaling pathway, thereby enhancing the intestinal mechanical and immune barriers. However, the current study primarily emphasized the overall effects of microorganisms, but the specific roles of individual microbial species in mitigating intestinal injury remain unclear. Future studies should further investigate these roles by cultivating key bacterial genera in vitro. Owing to the co-metabolism existing between the host and gut flora, this study failed to validate the origin of metabolites; the follow-up experiments will clarify the source of differential metabolites through co-culture of fecal bacterial solution in vitro. While our 28-day pretreatment model demonstrates acute protection, long-term LTA supplementation warrants cautious optimism. It should be emphasized that the observed protective effect does not negate the primary role of abstinence. Instead, our findings suggest that LTA can be used as an adjunct additive to prevent accidental alcoholism.

## 5. Conclusions

LTA improved intestinal permeability, relieved intestinal tissue injury, and reduced intestinal inflammation and oxidative stress. It helps maintain intestinal flora homeostasis by increasing the relative abundance of *Alloprevotella*, *Candidatus_Saccharimonas*, *Muribaculum*, and *Prevotellaceae_UCG-001*. LTA also decreases LPS levels and boosts levels of the beneficial metabolites oxoglutaric acid and L-ascorbic acid. Furthermore, LTA upregulated HIF-1α, claudin-1, occludin, and ZO-1 and downregulated TLR4, NF-KB, PHD2, TNF-α, and IFN-γ at both the mRNA and protein levels. This modulation occurs through regulation of the TLR4/NF-κB/HIF-1α axis via the HIF-1 signaling pathway, reinforcing both the mechanical and immune barriers of the intestine. These mechanisms collectively alleviate intestinal damage induced by acute alcohol consumption. In summary, these results suggest that LTA has the potential to protect against acute alcoholic intestinal injury and provide a scientific basis for LTA as a dietary supplement.

## Data Availability

The original contributions presented in this study are included in the article/Supplementary Materials. Further inquiries can be directed to the first author.

## Supplementary Materials

The following supporting information can be downloaded at: https://www.mdpi.com/article/10.3390/nu17040720/s1, Figure S1. Evolution of body weight and food intake of mice during the course of the feeding. Figure S2. KEGG map of HIF-1 signaling pathway. Table S1. Histological scoring criteria. Table S2. Related gene primer sequences.
