# Supplementary material for: L-Theanine Mitigates Acute Alcoholic Intestinal Injury by Activating the HIF-1 Signaling Pathway to Regulate the TLR4/NF-κB/HIF-1α Axis in Mice
Source: Nutrients. 2025 Feb 18;17(4):720. doi: 10.3390/nu17040720 (PMC11857980; doi:10.3390/nu17040720)
Supplement: Supplementary file 1 [file nutrients-17-00720-s001.zip › nutrients-3470261-supplementary.pdf]

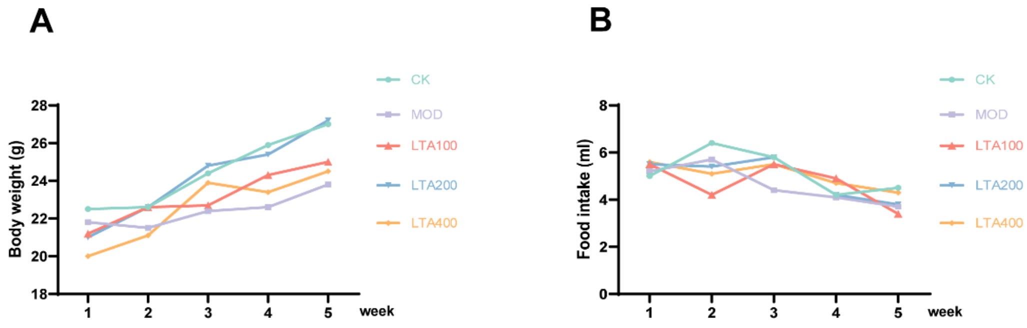

Figure S1. Evolution of body weight and food intake of mice during the course of the feeding.

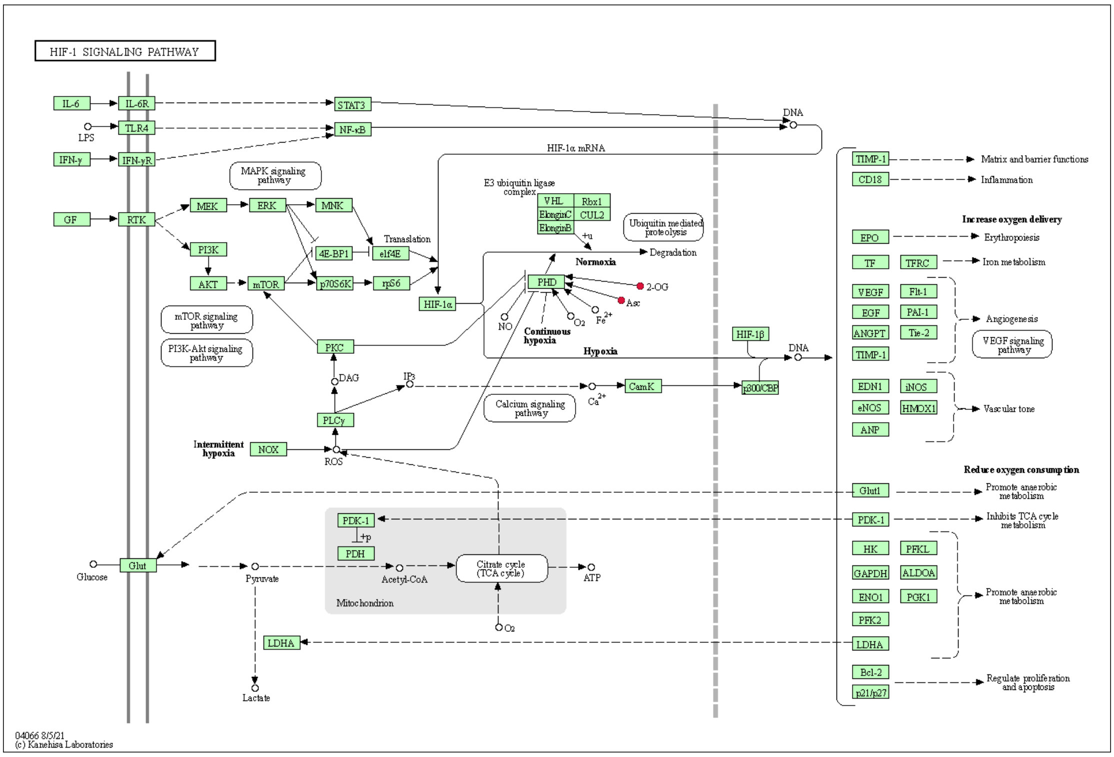

Figure S2. KEGG map of HIF-1 signaling pathway

Table S1 Histological scoring criteria

scroe	inflammation	lymphoid tissue	intestinal villus	Intestinal gland
0	none	none	none	none
1	occasional	occasionally seen lymphoid follicles	distorted and deformed intestinal villi	irregular morphology of intestinal glands
2	inflammation of submucosa cells	mild hyperplasia of lymphatic tissue	shortening of small intestinal villi	reduced number of intestinal glands
3	inflammation of the submucosa and lamina propria	severe hyperplasia of lymphatic tissue	large amount of intestinal villi becoming shorter	
4	extensive inflammation of the submucosa and lamina propria		intestinal villus autolysis	

Table S2 Related gene primer sequences

Gene Name	Forward nucleotide sequence primers (5'-3')	Reverse nucleotide sequence primers (5 '-3')
HIF-1α	GGATGAGTTCTGAACGTCGAAA	GGGGAAGTGGCAACTGATGA
PHD	TTACCCAGGCAACGGAACAG	CAGTTATTGCGTACCTTGGCG
TLR4	GGACTCTGATCATGGCACTGT	GGAATGTCATCAGGGACTTTGC
NF-κB	GCATTCTGACCTTGCCTATCT	CTCCAGTCTCCGAGTGAAGC
Claudin1	AGCTGTGCATGGCCTCTTGT	CCAATGTCAATGGCAACACC
Occludin	TGGCAAAGTGAATGGCAAGC	TCATAGTGGTCAGGGTCCGT
ZO-1	GAGCCCCCTAGTGATGTGTG	AGACCAACCGTCAGGAGTCA
IFN-γ	TGAACGCTACACACTGCATCT	GTTGCTGATGGCCTGATTGTC
TNF-α	CCCTCACACTCACAAACCAC	ACAAGGTACAACCCATCGGC
β-actin	GGCTGTATTCCCTCCATCG	CCAGTTGGTAACAATGCCATG
